# Holding all the CARDs: Quinone-induced oxidation and phosphorylation of GRXC1 impacts root growth

**DOI:** 10.1093/plphys/kiaf450

**Published:** 2025-09-27

**Authors:** Anna Moseler

**Affiliations:** Assistant Features Editor, Plant Physiology, American Society of Plant Biologists; INRES-Chemical Signalling, University of Bonn, Bonn 53117, Germany

Given their sessile lifestyle, plants utilize a sophisticated set of perception mechanisms for adjusting to an ever-changing environment. Perceived triggers can be abiotic, such as light, drought, and salinity, or biotic—from the plant itself or from other interacting species. Regarding other species, specific signals can be mediated by biologically active compounds, called allelochemicals, that are produced by plants to influence plant–plant, plant–insect, and plant–microbe interactions (allelopathy) (Bais et al. 2003). Allelochemicals are a subset of secondary metabolites and include quinones, which are widely synthesized redox-active molecules (Hillion and Antelmann 2015; Widhalm and Rhodes 2016; Bolton and Dunlap 2017). In Arabidopsis, the quinone 2,6-dimethoxy-1,4-benzoquinone (DMBQ) is perceived by the membrane-bound receptor CANNOT RESPOND TO DMBQ 1 (CARD1) (Laohavisit et al. 2020). CARD1 is a leucine-rich repeat receptor-like kinase and is identical to HYDROGEN-PEROXIDE-INDUCED CA^2+^ INCREASES 1 (HPCA1), which was identified in the same year as an extracellular sensor of hydrogen peroxide (H_2_O_2_) (Wu et al. 2020). The receptor has an extracellular domain that protrudes into the apoplast—the compartment outside of a plant cell's plasma membrane and an intracellular kinase domain. In both studies, it was shown that four cysteine residues in the extracellular domain are sites of quinone and H_2_O_2_ sensing triggering kinase activity and subsequently cytosolic calcium fluxes and stress-related responses (Laohavisit et al. 2020; Wu et al. 2020; Fichman et al. 2022). Although the molecular events that underlie perception of quinone signaling were addressed before (Laohavisit et al. 2020; Wu et al. 2020), the precise downstream signaling remains unclear as well as the relationship between quinone-induced cysteine modifications and the quinone–CARD1 signaling pathway.

In this issue of *Plant Physiology*, Liang Zhang and colleagues (Zhang et al. 2025) addressed this gap and analyzed the DMBQ-induced redox changes of the cysteine proteome and further determined the impact of phosphorylation on downstream factors by CARD1.

In an initial experiment, the authors dissected the oxidative response in Arabidopsis roots to DMBQ. While the treatment with DMBQ elevated intracellular reactive oxygen species (ROS) levels in wild-type (WT) roots, DMBQ application failed to increase ROS production in *card1* roots. At the same time, treatment with DMBQ inhibited root growth of the WT but not of the *card1* mutant. Further studies on the impact of DMBQ on the NADPH oxidase mutant *rbohD* (RESPIRATORY BURST OXIDASE PROTEIN HOMOLOG D, Torres et al. 2002), a key enzyme for ROS production, showed that ROS accumulation is required for the DMBQ-induced inhibition of root growth. These data indicate that DMBQ induces accumulation of ROS via CARD1 and RBOHD resulting in an inhibition of root growth.

In the next step, Zhang and colleagues investigated the effect of DMBQ on the redox status of Arabidopsis proteins. Using selective probes for thiols (Cys-SH), sulfenic acids (Cys-SOH), or sulfinic acids (Cys-SO_2_H), respectively, they identified 3,584 Cys-SH sites mapping to 2,349 proteins; 2,350 Cys-SOH sites corresponding to 1,583 proteins; and 17 Cys-SO_2_H sites from 17 proteins in DMBQ-treated WT plants. Comparison with untreated plants revealed that 733 proteins were significantly oxidized in response to DMBQ. Analysis of an earlier redox proteome dataset obtained after H_2_O_2_ treatment (Huang et al. 2019) highlighted both overlap and differences in oxidized proteins: 82% of the overlapping proteins exhibited the same Cys-SOH site, indicating that DMBQ and H_2_O_2_ treatments had similar effects on protein oxidation. Functional enrichment analyses of the proteins specifically modified upon the respective treatment suggested distinct biological impacts: DMBQ-modified proteins were associated with protein folding and redox homeostasis, while H₂O₂-targeted proteins were involved in RNA-related processes. Further analysis of proteins that were specifically oxidized upon DMBQ-treatment pointed to biological processes tied to redox homeostasis, root development, and responses to cold stress—consistent with the phenotypic observation that DMBQ suppresses root elongation.

To probe the regulatory role of CARD1, the authors performed comparative redox proteomics in the *card1* mutant. After DMBQ treatment, the number of oxidized proteins was markedly reduced compared with WT, suggesting CARD1 as a key modulator of DMBQ-triggered protein oxidation. To explore the oxidized proteins regulated by CARD1 in response to DMBQ treatment, Zhang and colleagues compared the sets of oxidized proteins after DMBQ treatment in both the WT and *card1*. They identified 237 proteins whose oxidation was dependent on CARD1 activation by DMBQ, with many localizing to the cytoplasm, chloroplast, or plasma membrane. Conversely, 133 proteins exhibited oxidation only in the DMBQ-treated *card1* mutant, suggesting that CARD1 may also act as a repressor of redox modification in certain contexts. These findings suggest a dual regulatory model for CARD1, both promoting and restricting protein oxidation.

In the last part of their study, Zhang and colleagues dissect the downstream factors of the DMBQ-CARD1 signaling pathway. Using immunoprecipitation-coupled mass spectrometry, the authors identified several candidate CARD1-interacting proteins, including those oxidized in response to DMBQ treatment. Among these, GLUTAREDOXIN C1 (GRXC1), a small oxidoreductase that was oxidized at its catalytic Cys (C39) residue upon DMBQ treatment, emerged as a CARD1 interactor especially in its dimer form (Fig. 1). The GRXC1 dimer was detected previously in planta and in vitro assays showed that the dimer can coordinate an iron-sulfur (FeS) cluster via the catalytic cysteines being less active than the monomer that lacks the FeS cluster (Riondet et al. 2012). A combination of luciferase complementation, bimolecular fluorescence complementation, and co-immunoprecipitation assays confirmed the CARD1–GRXC1 interaction in planta, primarily localizing to the plasma membrane. This is in line with a previous study that identified GRXC1 as a cytosolic protein attached to membranes through myristoylation (Schlößer et al. 2024). Additionally, Zhang and colleagues showed that CARD1 also directly phosphorylates GRXC1 at threonine 55. Substitution of the redox-sensitive C39 residue diminished GRXC1 phosphorylation, implicating the redox status in controlling phosphorylation. Furthermore, a phosphomimic variant of GRXC1 (T55D) exhibited decreased binding to CARD1, while a nonphosphorylatable variant (T55A) maintained the interaction. This highlights a potential feedback loop in which redox status and phosphorylation influence GRXC1–CARD1 complex formation. DMBQ treatment in *Nicotiana benthamiana* leaves elevated GRXC1 phosphorylation in a CARD1-dependent manner, further reinforcing the idea that CARD1 serves as both a redox and phospho-relay hub. RNA-seq profiling of Arabidopsis WT, DMBQ-treated WT, *card1*, and *grxc1* mutants revealed strong transcriptomic overlap among the two mutants and DMBQ-treated WT, with hundreds of differentially expressed genes shared and enriched in stress-related pathways, particularly immune responses and oxidative stress. These findings point to a common DMBQ-CARD1-GRXC1 signaling module. Physiologically, both *card1* and *grxc1* mutants exhibited attenuated root growth inhibition upon DMBQ exposure. Complementation with WT CARD1 or GRXC1 restored sensitivity, whereas an inactive (C39S) or nonphosphorylatable (T55A) GRXC1 variant failed to fully complement, highlighting the dual importance of redox modification and phosphorylation for GRXC1 function.

**Figure 1.**
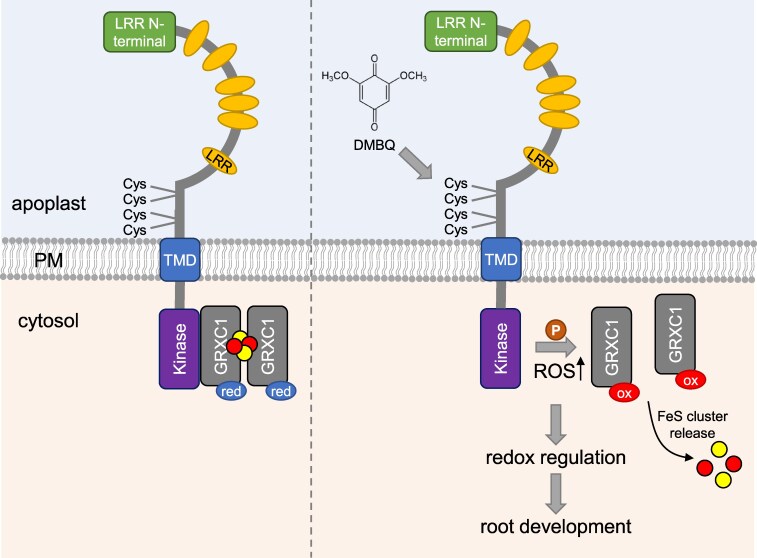
The DMBQ-CARD1-GRXC1 signaling pathway. The quinone DMBQ is perceived by the leucine-rich repeat receptor-like kinase CARD1 resulting in cysteine modifications in the ectodomain. The cysteine modification results in phosphorylation of GRXC1 and subsequent oxidation of the active site cysteine. GRXC1 in its reduced form is able to form a dimer coordinating an FeS cluster. Because the cluster is ligated by the catalytic cysteines the cluster-bridged dimer is enzymatically inactive (Riondet et al. 2012). Upon oxidation, the FeS cluster is released resulting in monomerization and activation of GRXC1. Upon activation, GRXC1 could facilitate the regulation of specific protein targets necessary for root development (modified after Zhang et al. 2025). Cys, cysteine; LRR, leucine-rich repeat motif; ox, oxidized; P, phosphorylation; PM, plasma membrane; red, reduced; TMD, transmembrane domain.

Overall, the study by Zhang and colleagues revealed how CARD1 phosphorylates the redox-sensitive GRXC1, likely triggering a dimer-to-monomer transition that modulates GRXC1 activity and downstream signaling. This dual-modification mechanism ensures tight regulation of gene expression and subsequently root growth, providing a striking example of how plants couple redox and phospho-signaling to respond to environmental cues. This work not only deepens our understanding of quinone signaling in plants but also highlights the importance of multi-modal regulation by posttranslational modifications at the protein level. Further studies are needed, however, to unravel the precise function of GRXC1 and its interaction partners. Notably, GRXC1 is present exclusively in eudicots (Couturier et al. 2009), where the phosphorylated T is also highly conserved (Schlößer et al. 2024). The integration of redox and phosphorylation control by the CARD1–GRXC1 module may represent a specific strategy by which eudicots fine-tune responses to dynamic environments.

## Data Availability

No new data included in this article.

## References

[kiaf450-B1] Bais HP, Vepachedu R, Gilroy S, Callaway RM, Vivanco JM. Allelopathy and exotic plant invasion: from molecules and genes to species interactions. Science. 2003:301(5638):1377–1380. 10.1126/science.1083245 12958360

[kiaf450-B2] Bolton JL, Dunlap T. Formation and biological targets of quinones: cytotoxic versus cytoprotective effects. Chem Res Toxicol. 2017:30(1):13–37. 10.1021/acs.chemrestox.6b00256 27617882 PMC5241708

[kiaf450-B3] Couturier J, Jacquot JP, Rouhier N. Evolution and diversity of glutaredoxins in photosynthetic organisms. Cell Mol Life Sci. 2009:66(15):2539–2557. 10.1007/s00018-009-0054-y 19506802 PMC11115520

[kiaf450-B4] Fichman Y, Zandalinas SI, Peck S, Luan S, Mittler R. HPCA1 is required for systemic reactive oxygen species and calcium cell-to-cell signaling and plant acclimation to stress. Plant Cell. 2022:34(11):4453–4471. 10.1093/plcell/koac241 35929088 PMC9724777

[kiaf450-B5] Hillion M, Antelmann H. Thiol-based redox switches in prokaryotes. Biol Chem. 2015:396(5):415–444. 10.1515/hsz-2015-0102 25720121 PMC4438307

[kiaf450-B6] Huang J, Willems P, Wei B, Tian C, Ferreira RB, Bodra N, Martínez Gache SA, Wahni K, Liu K, Vertommen D, et al Mining for protein S-sulfenylation in Arabidopsis uncovers redox-sensitive sites. Proc Natl Acad Sci U S A. 2019:116(42):21256–21261. 10.1073/pnas.1906768116 31578252 PMC6800386

[kiaf450-B7] Laohavisit A, Wakatake T, Ishihama N, Mulvey H, Takizawa K, Suzuki T, Shirasu K. Quinone perception in plants via leucine-rich-repeat receptor-like kinases. Nature. 2020:587(7832):92–97. 10.1038/s41586-020-2655-4 32879491

[kiaf450-B8] Riondet C, Desouris JP, Montoya JG, Chartier Y, Meyer Y, Reichheld JP. A dicotyledon-specific glutaredoxin GRXC1 family with dimer-dependent redox regulation is functionally redundant with GRXC2. Plant Cell Environ. 2012:35(2):360–373. 10.1111/j.1365-3040.2011.02355.x 21767278

[kiaf450-B9] Schlößer M, Moseler A, Bodnar Y, Homagk M, Wagner S, Pedroletti L, Gellert M, Ugalde JM, Lillig CH, Meyer AJ. Localization of four class I glutaredoxins in the cytosol and the secretory pathway and characterization of their biochemical diversification. Plant J. 2024:118(5):1455–1474. 10.1111/tpj.16687 38394181

[kiaf450-B10] Torres MA, Dangl JL, Jones JDG. Arabidopsis gp91^phox^ homologues *AtrbohD* and *AtrbohF* are required for accumulation of reactive oxygen intermediates in the plant defense response. Proc Natl Acad Sci U S A. 2002:99(1):517–522. 10.1073/pnas.012452499 11756663 PMC117592

[kiaf450-B11] Widhalm JR, Rhodes D. Biosynthesis and molecular actions of specialized 1,4-naphthoquinone natural products produced by horticultural plants. Hortic Res. 2016:3(1):16046. 10.1038/hortres.2016.46 27688890 PMC5030760

[kiaf450-B12] Wu F, Chi Y, Jiang Z, Xu Y, Xie L, Huang F, Wan D, Ni J, Yuan F, Wu X, et al Hydrogen peroxide sensor HPCA1 is an LRR receptor kinase in Arabidopsis. Nature. 2020:578(7796):577–581. 10.1038/s41586-020-2032-3 32076270

[kiaf450-B13] Zhang L, Wang L, Qiang X, Chen K, Xue J, Liu Z, Sun L, Zhao Z, Bai L, Yu F, et al The CARD1–GLUTAREDOXIN C1 module regulates Arabidopsis root growth via quinone-induced oxidation. Plant Physiol. 2025; 10.1093/plphys/kiaf419 40991720

